# Contrasting Lesion Dynamics of White Syndrome among the scleractinian corals *Porites* spp

**DOI:** 10.1371/journal.pone.0129841

**Published:** 2015-06-29

**Authors:** Paula Lozada-Misa, Alexander Kerr, Laurie Raymundo

**Affiliations:** 1 NOAA Pacific Islands Fisheries Science Center, Coral Reef Ecosystem Division, Honolulu, Hawaii, United States of America; 2 Marine Laboratory, University of Guam, Guam, United States of America; King Abdullah University of Science and Technology, SAUDI ARABIA

## Abstract

White syndrome (WS) is currently the most prevalent disease of scleractinian corals in the Indo-Pacific region, with an ability to exist in both epizootic and enzootic states. Here, we present results of an examination of WS lesion dynamics and show that potentially associated traits of host morphology (i.e., branching vs. massive), lesion size, and tissue deposition rate influence disease severity and recovery. Lesion healing rate was positively correlated with initial lesion size in both morphologies, but the rate at which lesions healed differed between morphologies. New lesions in branching *Porites cylindrica* appeared less frequently, were smaller and healed more quickly, but were more abundant than in closely-related massive *Porites* sp(p). The positive association between lesion size and healing rate was partly explained by geometry; branching limited lesion maximum size, and larger lesion margins contained more polyps producing new tissue, resulting in faster healing. However, massive colonies deposited tissue more slowly than branching colonies, resulting in slower recovery and more persistent lesions. Corallite size and density did not differ between species and did not, therefore, influence healing rate. We demonstrated multiple modes of pathogen transmission, which may be influenced by the greater potential for pathogen entrainment in branching vs. massive morphologies. We suggest that attributes such as colony morphology and species-specific growth rates require consideration as we expand our understanding of disease dynamics in colonial organisms such as coral.

## Introduction

Reports continue to place “white diseases” as the most prevalent coral disease state across the global range of coral reefs and scleractinian taxa, constituting a significant cause of coral decline [1–7]. In the Indo-Pacific, the term *white syndrome* (WS) [8] has been adopted to refer to a suite of disease signs affecting diverse coral taxa, with the tacit acknowledgement that these signs may involve more than one causal agent and may manifest differentially among affected host taxa [9,10]. In general, white syndrome manifests as single to multiple lesions displaying a clear boundary between healthy tissue and recently denuded skeleton, and is characterized by rapid tissue loss that does not progress as a band [10–12]. Recent studies have suggested more than one putative cause; Sussman et al. [3] presented strong evidence implicating the *α* proteobacteria *Vibrio* spp. in widely dispersed outbreaks, while Ainsworth et al. [13] found no evidence of bacterial involvement, citing unexplained apoptosis as a cause of tissue death. Intriguingly, Work et al. [14] described a mechanism of tissue loss involving cell-lineage parasitism as a result of chimera formation during larval settlement and subsequent colony fusion. On the community level, the disease can manifest as chronic, low-level enzootics [15] or acute epizootics [16,3]. Prevalence on the Great Barrier Reef may possess a seasonal component, with higher prevalence in warmer months [17,6], while in Guam and Hawaii, no seasonality is apparent [4,18]. Water quality may influence etiology; sewage-based eutrophication was linked with disease severity in corals on Guam reef flats [19]. White syndrome affects at least eight genera from five families and is the most prevalent disease on Guam reefs; prevalence as high as 40% has been recorded [20,4].

Although our understanding of coral diseases expands with additional reports addressing etiology, environmental drivers and causation, little attention has been paid to the influence of colony morphology on the disease process. In colonial animals, morphology is the result of evolutionary trade-offs driven by limited energy resources. Sessile colonial animals must address the simultaneous challenges of food acquisition, growth, reproduction, and defense in variable environments. Coral phenotypic plasticity in response to spatially and temporally variable environmental drivers such as light and wave energy has been well documented [21,22] and Rinkevich [23] stressed the genetic basis for this plasticity. However, only recently has immunodefense been considered as a potential evolutionary driver of morphological characters in corals. Palmer et al. [24] hypothesized a trade-off between growth and immune function in corals with contrasting life histories. *Porites*, a slow-growing genus known to have lower susceptibility to bleaching and disease, showed superior immune function to *Acropora*, which grows rapidly but is more susceptible to both. In corals, where the living tissue is a thin veneer covering a skeleton varying widely in structural complexity, colony morphology could play a role in disease susceptibility. For example, morphology could influence the frequency at which a colony comes in contact with either water-borne or vector-transmitted pathogens, how easily potential pathogens are sloughed off in the surface mucous layer, the success of vector-borne transmission, or the geometry of a disease-generated lesion. All of these attributes could have a profound influence on the overall impact of a disease on both colony and community scales.

This report provides preliminary evidence for a role of colony attributes—morphology and growth—on both colony-level and population-level disease impacts. Here, we report differential dynamics in WS in two closely related species with contrasting colony morphologies: branching *Porites cylindrica* and massive *Porites* spp. We examine the behavior of WS lesions within individual colonies and discuss this in the context of disease prevalence and severity in monitored populations in Guam, Micronesia.

## Materials and Methods

The Guam Dept. of Aquatic and Wildlife Resources (DAWR), under the Dept. of Agriculture, issued a permit to the UOG Marine Lab in 2001 to allow the ongoing collection of corals in Guam for education and research (Section 63123, Title 5, GCA). We reported all corals collected for the transmission study and corallite measurements to the DAWR Office. We did not collect from Marine Preserves. Institutional Animal Care and Use Committee (IACUC) regulations at UOG do not include invertebrates, so we did not need an IACUC permit to conduct this project. All other aspects of this project were conducted *in situ*, without removing or altering corals and, thus, did not require a permit on Guam.

### Gross lesion characterization and dynamics in contrasting colony morphologies

To monitor lesion behavior *in situ* on contrasting colony morphologies, we haphazardly selected 10 colonies of the branching coral *Porites cylindrica* and 10 colonies of massive *Porites* spp. (*P*. *lobata*, *P*. *australiensis* or *P*. *lutea;* field identification is unreliable with massive *Porites*; R. Randall, pers. comm.) in Luminao Reef, western Guam. Colonies selected were located at least 10 m apart, at 1.5 m to 2 m depth, and exhibited active WS lesions. Colony maximum diameter was measured, and five haphazardly selected lesions per colony were tagged, photographed and described following Work and Aeby [25], and their position on the colony noted. Tagged lesions were photographed monthly for size, shape and status, and all active lesions were counted per colony, to track disease severity (see below), from October 2009 to June 2010. Lesion size (cm^2^) was digitally measured from macro-photographs, using Image J (v.144) software (NIH 2010). In cases where lesions circumscribed a branch, macro-photographs were taken of the front and back of the branch and the sum of the front and back lesion measurements were summed to obtain total lesion size. As branches of *P*. *cylindrica* are laterally flattened, this method minimized discrepancies in size estimates due to curvature distortion in the photographs. Lesion status was described each month as *enlarging* (i.e., active tissue loss), *recovering* (tissue resheeting), or *in stasis* (neither tissue loss nor regrowth along the affected margin). Lesion recovery rate (cm^2^ d^-1^) was defined as the rate of lesion size reduction (via tissue resheeting) and calculated as the change in lesion size between two censuses divided by the number of days between census periods.

### Colony-scale differences in corallite structure

To test whether differences in corallite size or distribution could explain lesion size or growth rate within a colony, we tested for significant differences in corallite density and size between colony morphologies. Ten clinically healthy colonies of each of the two *Porites* morphologies, adjacent to the monitored colonies, were selected. On each colony, a healthy region or branch was photographed (using a Canon G10) next to a ruler for scale. Corallite properties were then examined digitally from the 10 macro-photographs of each growth form using Image J (v.144h) software. To avoid examining corallites from a curved area on the image, corallite density was quantified within a flat area bounded by a 0.50 cm^2^ grid. To test for differences in corallite size between the two growth forms, 20 corallites were randomly selected within the same 0.50 cm^2^ grid of each macro-photograph (n = 200 per morphology) and corallite diameters (cm) were measured across opposite ends of the outer corallite walls.

### Community-scale disease prevalence and severity

**Fig 1:**
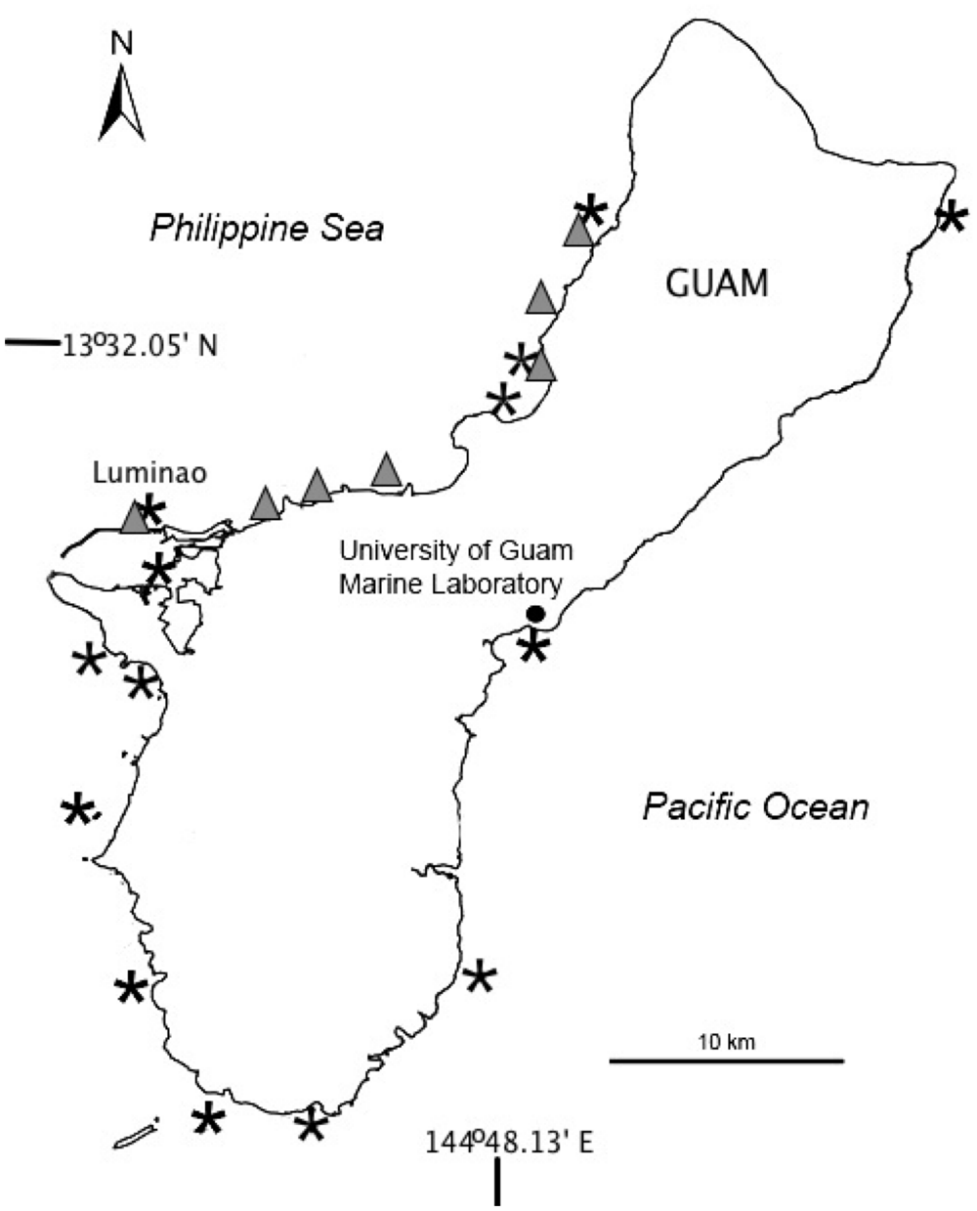
Map of Guam. Map shows the original survey sites to establish baseline disease levels on Guam (black stars) and the seven sites monitored for WS severity (grey triangles).

A total of fourteen reef flats and fore reefs around Guam (Fig 1) were surveyed between 2006 and 2008 to establish a reference assessment of prevalence of all described diseases affecting Guam corals [4]. Transects were placed parallel to shore within the zone with the highest coral density, at least 10m apart, at 1–3m depth on reef flats, and 3–10m depth on the forereef. All coral colonies with at least ½ of the colony falling within the belt were identified to the lowest taxon possible, and their health status noted, using Raymundo et al. (2008). Data on *Porites* spp white syndrome were extracted from these initial surveys, to examine prevalence of white syndrome in *Porites cylindrica* and *P*. *lobata/lutea*. Prevalence was calculated as # WS-infected colonies / Total # colonies censused * 100, per transect.

Disease severity was expressed via two metrics, to account for the large variation in lesion size and number: 1) as percent tissue loss per colony (% TL), and 2) as rate of appearance of new lesions (# of lesions colony^-1^ month^-1^, standardized to colony maximum diameter). To determine whether severity differed between morphologies, 10 colonies per morphology selected for monitoring in Luminao reef (see above) were also censused monthly for the number and size of active lesions for a period of seven months. We then compared the change in %TL from the initial to final censuses between the two morphologies. The rate of appearance of new lesions was calculated as the difference in lesion number between censuses and averaged per colony. To expand our sample size, additional data were extracted from another data set presented in Redding et al. [19], which monitored white syndrome severity using the same metrics (change in lesion size and number, standardized by colony maximum diameter) in populations of the same species (n = 71 colonies of *P*. *cylindrica* and n = 31 colonies of massive *Porites)*. These separate populations were located along permanent transects that were monitored for one year in seven reef flats along western Guam (refer to Fig 1).

### Modes of transmission

Possible pathways of transmission of the WS causative agent were investigated, as these could potentially be influenced by colony morphology. Using *P*. *cylindrica* branches 7–10 cm in length, two fragments with active WS lesions from each of eight donor colonies and 10 healthy fragments from each of eight clinically healthy donor colonies were removed using wire cutters from the Luminao reef flat (Collection License from Guam Dept. of Aquatic and Wildlife Resources, Section 63123, Title 5, GCA). Fragments were immediately transported in fresh seawater to the UOG ML for a laboratory transmission experiment. Sixteen aerated aquaria (9.5 l) filled with fresh seawater were randomly arranged in two water baths with eight aquaria per tank and maintained at ambient temperature (26–29°C) and light. Eight aquaria served as the experimental set-up with each aquarium containing four clinically healthy, non-clonal fragments and two diseased, non-clonal fragments. Two of the healthy fragments were placed in direct contact with a single lesion on each of the two diseased fragments and the two remaining healthy fragments were positioned at the opposite end of the aquarium. This allowed simultaneous assessments of both direct and waterborne transmission. Procedural control aquaria were set up the same way, but contained only clinically healthy fragments. All were monitored for WS lesion appearance for six weeks, with water changes every 3 d, during which fragments were photographed and checked for appearance and location of lesions. Lesion size was digitally measured from macro-photographs using Image J (v.144) to estimate lesion progression rates. Similar to the monitored lesions in the field, in cases where lesions circumscribed a branch, macro-photographs were taken of the front and back of the branch and the sum of the front and back lesion measurements were used to estimate lesion size.

### Data Analyses

Prior to performing statistical analyses, data were tested for normality and homoscedasticity using Shapiro-Wilks and Levene’s tests, respectively. Data were transformed when necessary to meet the assumptions of equal variance and normality for the use of parametric statistical tests. Nonparametric tests (Kruskall-Wallis and Mann-Whitney) were used if the data could not be transformed to meet assumptions of equal variance and normality. Statistical analyses of lesion data were performed in SPSS v.22.

To determine whether the number of lesions differed between the two morphologies, a Mann-Whitney *U* test was performed on lesion counts taken during the initial census in branching and massive colonies (n = 10). Natural log-transformed lesion sizes between the two morphologies and among colonies of each morphology were analyzed using a two-way nested ANOVA (colonies nested within morphology type) based on the measurements of tagged lesions (n = 5) recorded during the initial census period (n = 50 per morphology).

To analyze lesion dynamics as a function of colony morphology, lesion sizes were divided into five unequal size classes (A = healed, B = 0.1 to 10.9 cm^2^, C = 11 to 69.9 cm^2^, D = 70 to 1000 cm^2^). The size classes were constructed around naturally occurring breaks in the data set, to maximize accuracy in encompassing the wide range of lesion sizes from the pooled population of 100 lesions. The size classes presented here allowed us to illustrate important transitions of lesions that would otherwise be binned in one size class, while optimizing the number of bins used for ease in interpretation. Possible transitions between each size class included growth to the next size class, stasis (remaining within a size class), shrinkage to a smaller size class, or total disappearance (healing via resheeting). The probability of a lesion displaying each status was calculated per morphology, from the number of lesions experiencing each of these states within the 7-mo monitoring period. A Markov Chain was used to graphically portray these transitions [26,27]. Pearson Chi-square tests were used to compare differences between morphologies in the proportion of lesions in each (initial) size class and in the proportion of lesions in each (final) transition state.

To test for differences in the recovery rate of lesions between the two morphologies, we first used a one-way Analysis of Variance (ANOVA) tested for homogeneity of slopes, which were found to be different between morphologies (F_1,69_ = 19.312; *p*<0.0001). We then employed linear regression analyses per morphology to examine relationships between initial lesion size and natural log-transformed lesion recovery rate. To test whether corallite size and density varied between the two morphologies, a one-way ANOVA was performed on corallite density and a Kruskall-Wallis test was used to analyze corallite size differences. To compare differences in the monthly rate of appearance of new lesions between the two morphologies, we calculated the mean monthly rate of change in lesion number for each colony (n = 10) as the difference in the number lesions recorded between census periods. As we tested the mean colony rate of change across 7 mo to compare differences between morphologies, it was more appropriate to use a one-way ANOVA rather than a repeated measures ANOVA. Prior to analysis, a log (+10) transformation was applied to the data to meet the assumptions of normality and homoscedasticity.

White syndrome prevalence data collected from surveyed sites were examined using Kruskal-Wallis tests to compare prevalence between sites and morphologies. Regression analyses were used to assess relationships between lesion size and recovery rate and WS prevalence and colony density. The software package Data Desk was used for these analyses.

## Results and Discussion

### Gross lesion characterization in contrasting colony morphologies

**Fig 2:**
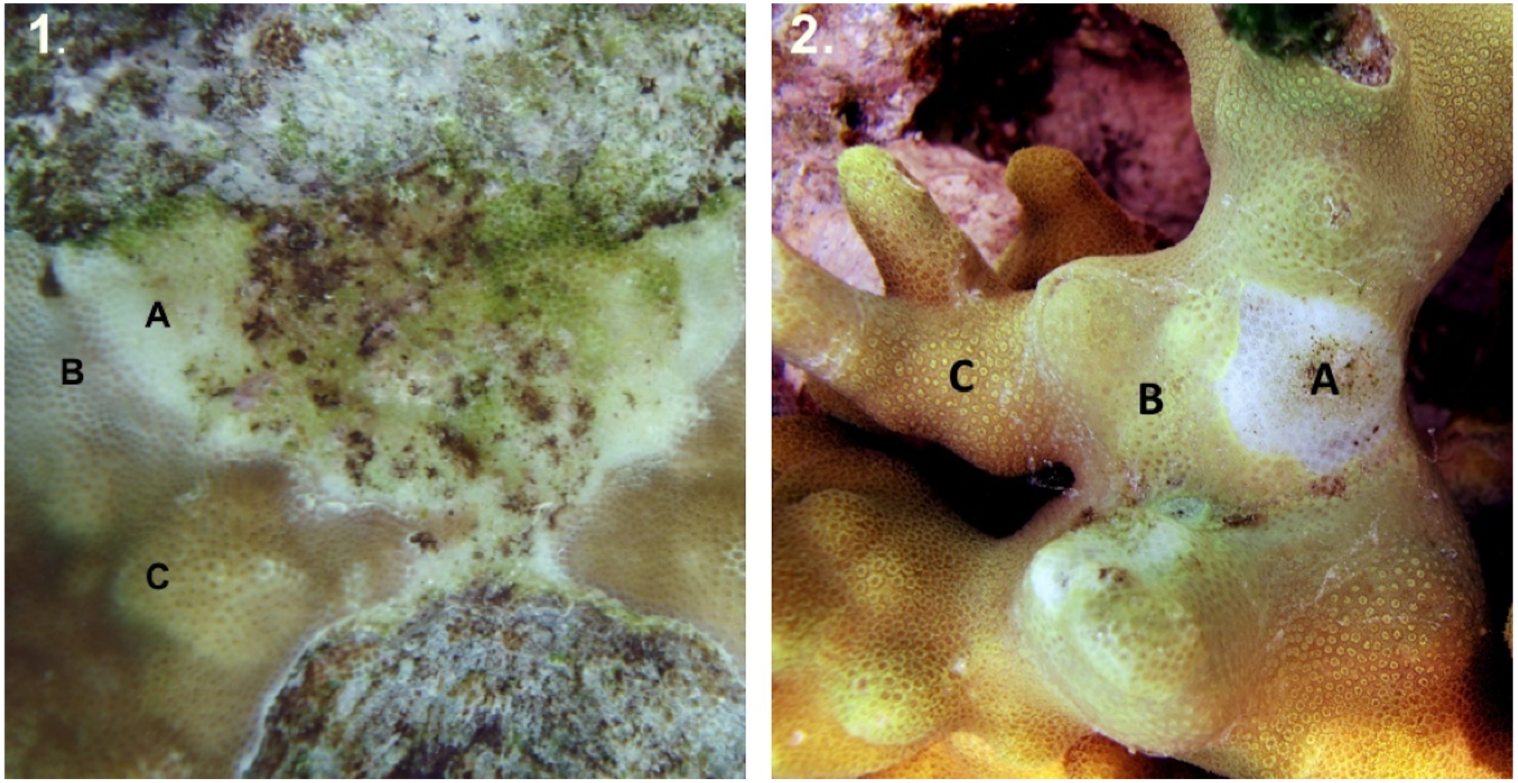
Typical white syndrome lesion on *Porites* spp. (A) zone of recent tissue loss and bare skeleton; (B) zone of progressing disease with excess mucous production, retracted polyps and discoloration; and (C) healthy tissue; **(1)** massive *Porites* sp(p).; **(2)** branching *Porites cylindrica*. Photo by L. Raymundo.

In both colony morphologies, a bleached zone characterized by excess mucous production, pale coloration and retracted polyps separated dead skeleton from healthy tissue along the disease front, suggesting that bleaching preceded tissue death (Fig 2). Lesions in branching *P*. *cylindrica* were small (range: 0.10–16.65 cm^2^; Table 1), while massive *Porites* spp. lesions were much larger (range: 0.42–976.71 cm^2^; Table 1). Lesion size was significantly different between morphologies (nested Two-Way ANOVA F_1,18_ = 32.291, *p* < 0.001), but not within colonies (F_18,80_ = 1.423, *p* = 0.144). Regardless of colony morphology, lesions displayed the same pattern of tissue loss and subsequent algal colonization.

**Table 1:** Summary of lesion dynamics in 20 monitored colonies on Luminao reef flat.

WHITE SYNDROME SEVERITY	*P*. *cylindrica*	massive *Porites* sp(p)
Mean no. lesions per colony per month	21± 1.74	18 ± 1.84
Mean lesion size (cm^2^)	2.55 ± 0.48	54.33 ± 1.60
Lesion size range (cm^2^)	0.10–16.65	0.42–976.71
Total healed lesions within 7 mo	43	20
Total lesions not healed within 7 mo	7	30

Colonies monitored for 7 mo; all values except mean no. of lesions col^-1^ mo^-1^ are based on n = 50 lesions per colony and n = 10 colonies; Mean ± SE.

Most lesions developed spontaneously without previous physical damage (70–90%), while a few (10%) enlarged from gastropod predation wounds *(Coralliophila violacea)* or physical damage, or initiated from a patch of bleached or discolored intact tissue (20%). Monitored lesions within colonies displayed all three described states: *enlarging*, with a zone of recently-exposed skeleton proximal to a zone of algal-colonized dead skeleton; *recovering*, with a clean, intact margin of resheeting tissue overgrowing bare skeleton or *stasis*, with an eroded margin, no new growth and skeleton colonized by turf algae, crustose coralline algae, or cyanobacteria.

### Lesion dynamics over time

**Fig 3:**
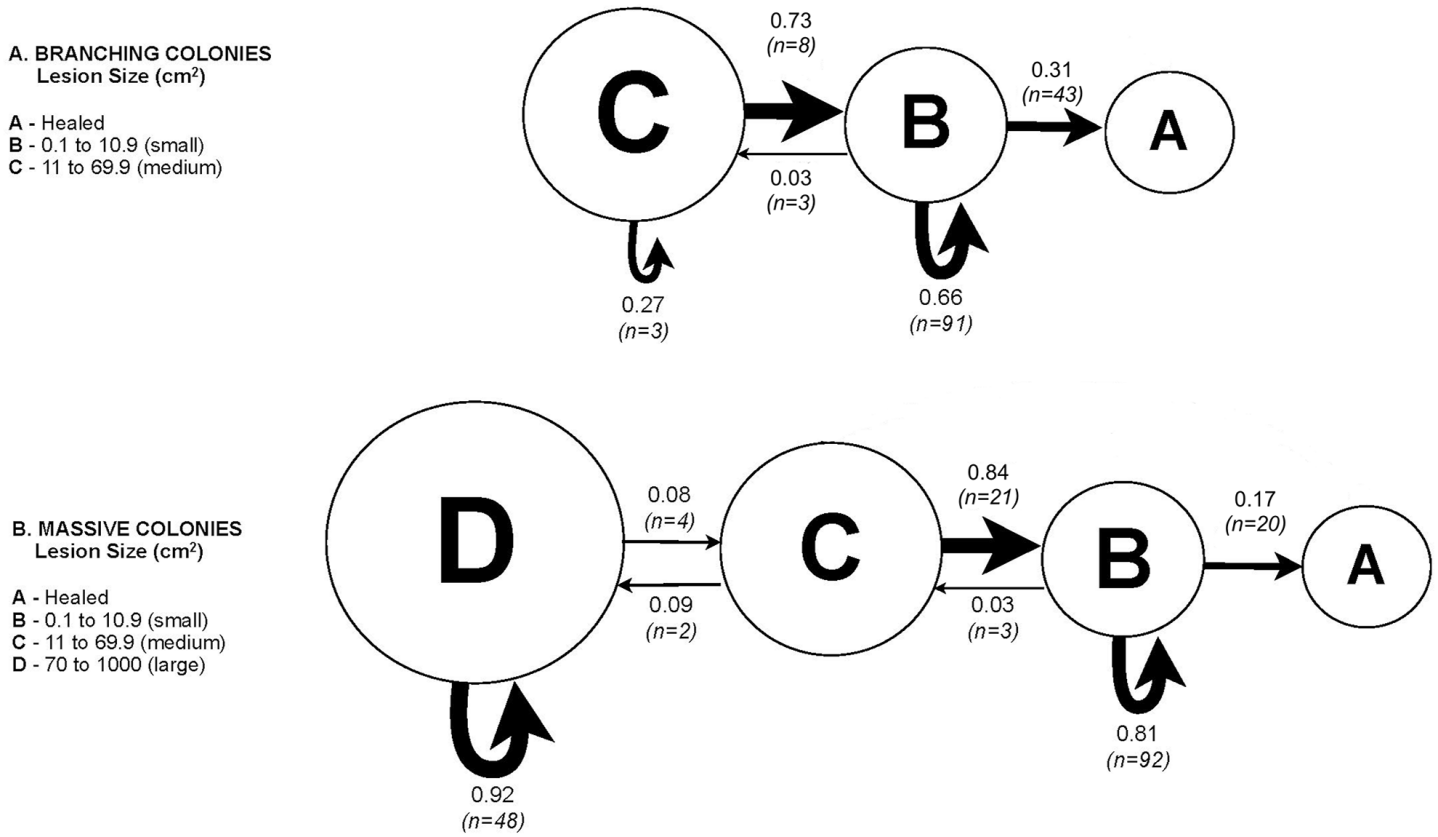
Markov Chain of probabilities for lesions on two morphologies transitioning between size classes. Size class C (for *Porites cylindrica;* branching colonies) and D (for *Porites* sp(p); massive colonies) represent the largest WS lesion size attained after 7 mo of census. WS lesion sizes ranged between 0.10–16.65 cm^2^ in branching colonies and 0.42–976.71 cm^2^ massive colonies.

The fates of monitored lesions of different sizes are presented in Fig 3 and S1 Table. The proportion of lesions in each state of transition significantly differed between branching and massive colonies (Pearson Chi-square test: χ^2^_(1, N = 100)_ = 9.013, *p* < 0.005). In branching colonies, most lesions averaged 5 cm^2^ (size class B). Over the 7-mo period, the majority of lesions (66%) remained unhealed but non-enlarging (stasis), while 31% resheeted. Only 3% enlarged to a maximum of 17 cm^2^ (size class C). These lesions often circumscribed the branch but then resheeted to size class B or A. In no monitored colonies were lesions observed to kill an entire branch or spread to adjacent branches. In massive colonies, initial lesions averaged 55 cm^2^, were highly variable in size and often orders of magnitude larger than those of branching lesions (Fig 3) (Pearson Chi-square test: χ^2^_(2, N = 100)_ = 32.775, *p*<0.001). The largest lesions rarely resheeted, persisting over time as bare skeleton colonized by algae. Some lesions in apparent stasis later “reactivated”, showing acute tissue loss and further disease progression.

**Fig 4:**
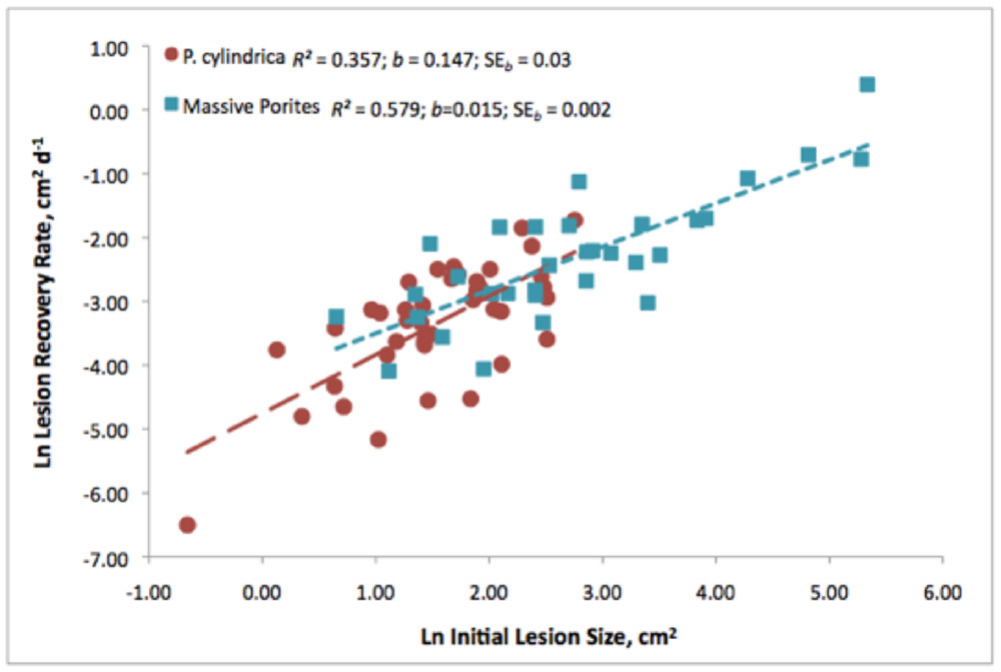
Regression of initial lesion size on lesion recovery rate. *Porites cylindrica* (red circles; n = 41 lesions from 10 colonies) and massive *Porites* (blue squares; n = 32 lesions from 10 colonies) in colonies monitored for ≤7 mo. Regressions based on untransformed X values, but logged here for clarity of presentation.

There was a strong positive relationship between lesion size and recovery rate in both branching and massive growth forms (linear regression; branching: *R* ^*2*^ = 0.357, F_1,39_ = 21.644, *p*<0.001; massive: *R* ^*2*^ = 0.579, F_1,30_ = 41.321, *p*<0.001) (Fig 4 and S2 and S3 Tables). Larger lesions resheeted at a faster rate than did smaller ones. Lesion geometry partially explains this observation; a longer linear margin around larger lesions results in more polyps actively producing tissue. However, there were also apparent species-related differences: lesions on massive colonies resheeted at a slower rate than did those on branching colonies (slope, branching: *b* = 0.147±SE_*b*_ = 0.03; slope, massive: *b* = 0.015±SE_*b*_ = 0.002; one-way ANOVA: F_1,71_ = 20.525, *p*< 0.0001). The morphology of massive colonies allows for larger surface areas across which lesions can continue to expand, whereas branches limit the available surface area for lesion expansion. We tested for equality of slopes between the two morphologies and found that they were significantly different (F_1,69_ = 19.312; *p*<0.0001), even after we reanalyzed our data after removing the largest lesions on the massive colonies from our analysis (*R* ^*2*^ = 0.195; F_1,24_ = 5.8; *p* = 0.02). This suggests additional influences on healing, such as species-specific rates of tissue deposition (mean linear growth in *P*. *lutea* = 0.02mm d^-1^ and mean skeletal extension rate in *P*. *cylindrica* = 2.7mm d^-1^) [28,29]. Slower growth in massive *Porites* helps to explain both the wide variability in lesion size and the persistence of large lesions.

In a branching morphology, access to lesions by corallivores that favor damaged tissue [30,31] is hampered, which would promote tissue regrowth. In a massive morphology, healing is hampered by both algal overgrowth and corallivory along lesion borders. Our observations suggested that lesions with a minimum surface area roughly 70 cm^2^ of tissue loss did not heal and algal colonization rate exceeded tissue growth rate (Fig 3; lower boundary of size class D). This is considerably larger than the 130 mm^2^ “closable” lesion size reported for massive *Montastrea annularis* [32], but is consistent with their findings of persistence of larger lesions on massive morphologies. Additionally, corallivory on lesion borders can slow disease progress in at least one band disease [33], and this effect would operate more effectively along large and exposed margins.

Roff et al. [10] observed that energy allocated to healing in *Acropora* was translocated toward lesions caused by physical injury, but away from WS lesions. The authors hypothesized that this could be a manifestation of “shut down reaction” [34], wherein diseased tissue is isolated from that of healthy, and energy is devoted to tissue most likely to heal. This phenomenon could explain lesions we considered to be “in stasis”. If pathogens entered a dormant state, they could persist as a latent infection, preventing healing but not enlarging. Our monitoring observations revealed that new WS lesions in massive *Porites* were often located along borders of persistent bare skeleton and lesions in stasis sometimes “reactivated”, consistent with what might be predicted from dormant pathogens. Alternatively, persistent bare skeleton patches could attract and harbor foreign material containing potential pathogens and, as such, chronically expose border tissue to infectious agents. Such phenomena would facilitate an enzootic state of disease within a population.

### Mechanisms of transmission

Lesions grossly identical to those observed *in situ* developed in four out of 16 healthy fragments (25%) in the direct contact experiment, and one out of the 16 (6.25%) free-standing fragments. On three out of the four direct contact fragments, lesions appeared directly at the point of contact, with one developing additional lesions resulting in 90% tissue loss by the end of the experiment. On the fourth direct contact fragment, the lesion appeared around a previous injury site. All lesions developed within 6 d of exposure and progressed at a mean rate of 6.9 mm^2^ d^-1^ for the length of the experiment. All fragments in the healthy control aquaria remained lesion-free throughout the experiment.

Thus, both direct contact and waterborne transmission were possible, though direct contact was more successful. A branching morphology could facilitate transmission by temporarily entraining water within colony branches and extending the residence time of pathogens in contact with coral tissue. This is consistent with our observation of more frequent appearance of lesions in branching colonies, many of which were not limited to external branches. Massive morphologies would be less influenced by this hydrodynamic regime, though we did not test for length of time needed for lesion development between the two morphologies. Further, massive *Porites* are known to periodically slough off mucous sheets [35], which serves to remove surface-adhering debris and bacteria. This process could also transmit pathogens to nearby colonies, with segments of mucous sheets more likely to become entangled within branching morphologies.

### Colony-scale differences in corallite size and distribution

Corallites in branching and massive morphologies did not differ in either density (*P*. *cylindrica*: 34.3±0.94 cm^-2^; *P*. *lobata/lutea*: 33.9±0.62 cm^-2^; F_1,18_ = 0.125, p = 0.728; S4 Table) or diameter (*P*. *cylindrica*: 0.12±0.0 cm; *P*. *lobata/lutea*: 0.12±0.00 cm; F_1,398_ = 0.00, p = 0.957; S4 Table). Therefore, we concluded that there was no evidence that the corallite features we assessed were differentially influencing disease dynamics in these species.

### Community-scale severity and prevalence in contrasting morphologies

**Fig 5:**
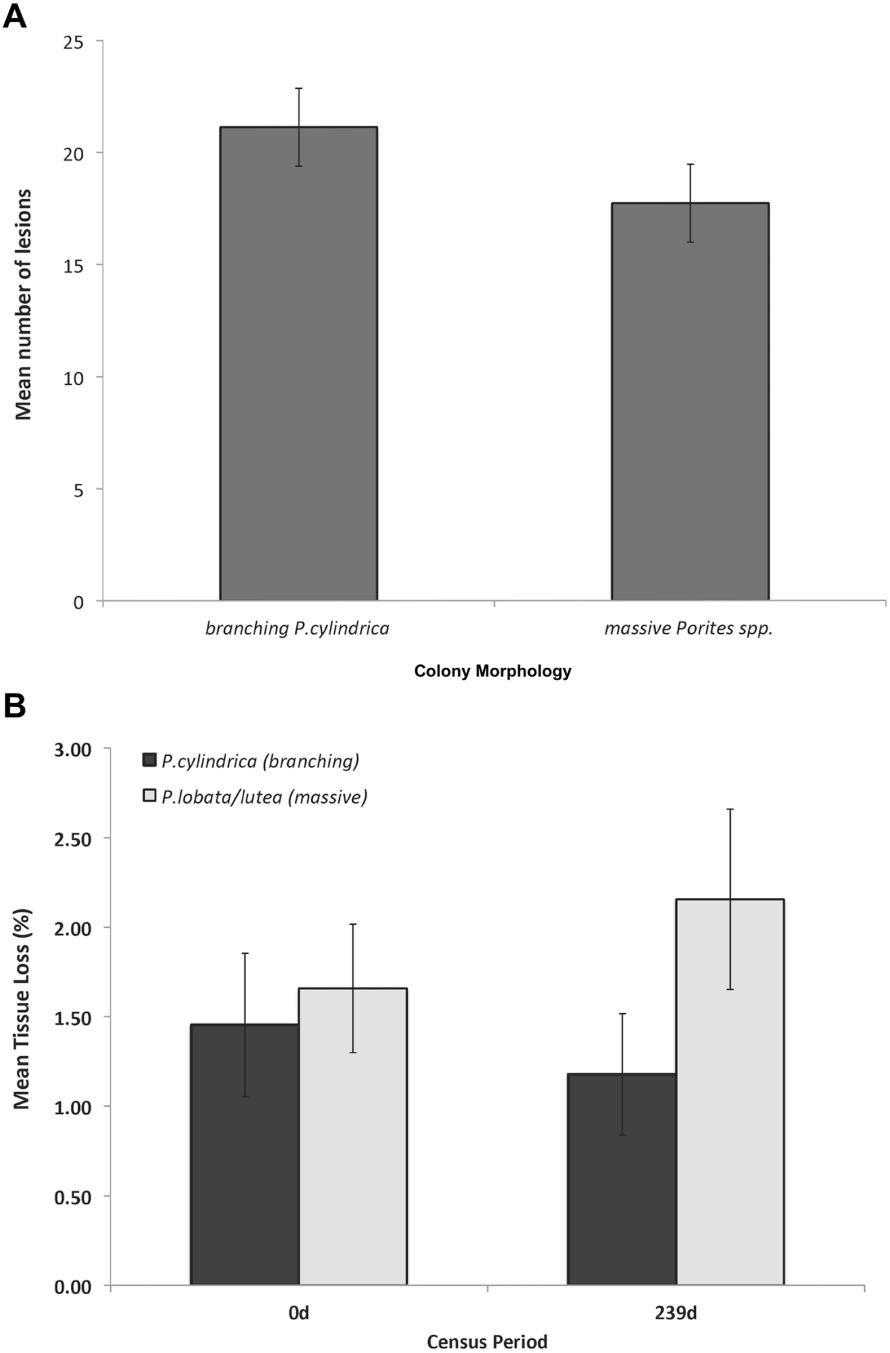
Measures of severity of WS in two colony morphologies (n = 10 colonies per morphology). (A) Mean (±SE) number of lesions per colony, averaged over a 7-mo monitoring period, in massive *Porites* vs. *Porites cylindrica* colonies, Luminao reef; (B) Comparison of mean percent tissue loss at beginning (T = 0) and end (T = 239 d) of the monitoring period in contrasting morphologies.

Lesions were significantly more numerous in branching *P*. *cylindrica* colonies than in massive colonies (Mann-Whitney *U* = 17.00, p < 0.05) (Table 1 and Fig 5A) while the rate of appearance of new lesions was significantly higher in the massive colonies than in the branching colonies (F_1,18_ = 8.804, p < 0.05). Smaller lesions on branching colonies, however, healed more successfully (86%), in contrast to those on massives (42%) (Table 1). Thus, monthly tissue loss, which is a measure of disease severity, differed between morphologies (F_1,18_ = 4.106, *p* < 0.05). More rapid healing resulted in decreased disease severity (by 28%) in branching colonies, while severity increased by 50% and accumulated over time in massive colonies (Fig 5B).

Prevalence of WS varied greatly between sites assessed in the baseline surveys (Kruskal-Wallis T_corr_ = 27.07; *p*_*corr*_ = 0.05; S5 Table) but did not vary between species (T_corr_: 0.01; *p*_*corr*_ = 0.9167; S5 Table). Prevalence was not strongly associated with colony density in either species (massive *Porites*: *R* ^*2*^ = 0.3; *P cyl*: *R* ^*2*^ = 0.28). In Guam, WS is an enzootic; prevalent year round, and affecting most of the common species on reef-flat communities [4,19]. Since it has been monitored, WS has never been observed to undergo an outbreak, as seen in Hawaii [16], Line Islands [5], Marshall Islands and Palau [3]. Epidemiological theory dictates that outbreaks are most probable at first exposure of a susceptible population, followed by an endemic state as susceptibility declines [36]. The decade-long chronic state of the disease on Guam suggests that it has been impacting corals prior to initial assessments initiated in 2005 and undetected outbreaks could have occurred prior to this date.

## Conclusions

We observed contrasting dynamics in white syndrome lesions in two common coral morphologies. Branching *P*. *cylindrica* displayed more lesions per colony than did massive *Porites* sp(p); a pattern consistent both spatially and temporally. However, *P*. *cylindrica* lesions were smaller and healed faster and more completely, whereas massive colonies had much larger lesions that persisted without healing. Irregardless of morphology or cause of tissue loss, larger lesions healed more quickly; lesion perimeter largely determined the amount of tissue regenerated [37,38]. However, colony morphology can influence the amount of tissue lost in the disease process; a branching morphology appears to limit the maximum size a lesion can become more effectively than a flat or massive surface area.

Additional influences on lesion behavior which must be considered are interactions between the causative agent and host. It is possible that our results were the product of different pathogens acting differentially on our host species, representing two different diseases. While observable signs of disease were identical with the exception of lesion size, we did not verify that the causal agents were the same. Lesion dynamics may also be driven by differential host immunodefense capability [24]. Although we chose congenerics for this study to minimize this possible effect, between-species differences in susceptibility is poorly investigated in the disease literature, yet might account for differences in progression rate and recovery success. However, we argue that in spite of these potential confounding factors, we present multiple lines of evidence (lesion behavior, corallite attributes, transmission mode, host tissue deposition rate, disease severity and prevalence) that consistently point to an influence of potentially associated traits of colony morphology and growth rate on coral disease dynamics.

Colony morphology and species-specific growth rate affect both short- and long-term dynamics of the disease. Further, WS spreads through multiple modes of transmission and chronically impacts dominant reef building species. Fighting a chronic infectious disease requires continual investment in healing and immunodefense; energy resources that may no longer be available for growth and reproduction. The impacts of chronic infection on reproductive output and on defense against other infections or stressors such as warming oceans have not been addressed, but should be examined. Taken together, these findings suggest the potential for subtle and persistent effects of white syndrome on coral communities. Given its prevalence throughout the Indo-Pacific, a better understanding of the causes and mechanisms of its persistence, as well as long-term effects on host colonies, would guide the development of disease management tools.

## Supporting Information

S1 Table Transition probabilities for lesion size dynamics. (XLSX)

S2 Table Lesion recovery rates per morphology. (PDF)

S3 Table Tissue loss rates, branching and massive morphologies. (XLSX)

S4 Table Corallite characteristics per morphology: density, diameter. (PDF)

S5 Table White syndrome baseline prevalence per site. (PDF)
