# Supplementary material for: Contrasting Lesion Dynamics of White Syndrome among the scleractinian corals Porites spp
Source: PLoS One. 2015 Jun 29;10(6):e0129841. doi: 10.1371/journal.pone.0129841 (PMC4488276; doi:10.1371/journal.pone.0129841)
Supplement: S2 Table — (PDF) [file pone.0129841.s002.pdf]

BRANCHING n=41 Init Size	resheet rate *P. cylindrica*	natural log Ln P cylindrica	MASSIVE n=32 Init Size	resheet rate massive *Porites*	natural log Ln Porites massive
**1.89**	0.013	-4.328661414	**123.26**	0.50	-0.702479293
**8.23**	0.042	-3.159559248	**207.03**	1.49	0.395692743
**3.00**	0.021	-3.840564041	**4.89**	0.03	-3.554607595
**2.05**	0.010	-4.65063256	**11.12**	0.16	-1.835035453
**0.52**	0.002	-6.502290171	**21.58**	0.11	-2.242332057
**6.81**	0.062	-2.780620894	**3.85**	0.06	-2.891548921
**5.63**	0.076	-2.57809663	**8.11**	0.16	-1.837914285
**2.78**	0.006	-5.164785974	**5.62**	0.07	-2.615417399
**4.05**	0.036	-3.33220451	**3.05**	0.02	-4.089704183
**2.83**	0.041	-3.183784505	**14.93**	0.16	-1.814686974
**12.30**	0.027	-3.593949719	**11.11**	0.05	-2.905784037
**6.63**	0.068	-2.688847994	**3.93**	0.04	-3.249966412
**8.25**	0.019	-3.985809175	**16.33**	0.32	-1.126130318
**4.18**	0.025	-3.684887433	**17.37**	0.07	-2.67735462
**3.26**	0.027	-3.629456034	**46.23**	0.18	-1.729786999
**15.70**	0.177	-1.729647359	**7.04**	0.02	-4.059005196
**4.31**	0.011	-4.556180799	**18.39**	0.11	-2.209238907
**6.62**	0.059	-2.830563796	**8.72**	0.06	-2.874498409
**3.54**	0.044	-3.131014469	**17.47**	0.11	-2.226444829
**2.62**	0.044	-3.131014469	**7.53**	0.06	-2.877060744
**1.14**	0.023	-3.760792036	**4.39**	0.12	-2.095570924
**6.42**	0.051	-2.975529566	**28.40**	0.17	-1.793406394
**9.88**	0.157	-1.851899515	**11.08**	0.06	-2.82294974
**3.59**	0.037	-3.298493453	**1.92**	0.04	-3.239495112
**5.41**	0.086	-2.459119564	**33.30**	0.10	-2.275751698
**12.30**	0.053	-2.941531817	**49.95**	0.18	-1.69957322
**11.83**	0.063	-2.769050685	**12.56**	0.09	-2.432946911
**4.43**	0.030	-3.513383862	**72.11**	0.34	-1.072061428
**4.69**	0.082	-2.498053922	**26.92**	0.09	-2.392720401
**3.64**	0.067	-2.69789783	**11.88**	0.04	-3.331702544
**4.12**	0.047	-3.063268481	**29.92**	0.05	-3.020299371
**7.45**	0.082	-2.498053922	**196.09**	0.46	-0.771719561
**1.90**	0.033	-3.421816669			
**6.28**	0.011	-4.526698571			
**11.94**	0.062	-2.77800879			
**5.31**	0.071	-2.644788004			
**11.69**	0.073	-2.613157122			
**7.71**	0.044	-3.121712076			
**10.77**	0.118	-2.135688007			
**4.18**	0.027	-3.606641356			
**1.42**	0.008	-4.803169556			
