# Supplementary material for: Contrasting Lesion Dynamics of White Syndrome among the scleractinian corals Porites spp
Source: PLoS One. 2015 Jun 29;10(6):e0129841. doi: 10.1371/journal.pone.0129841 (PMC4488276; doi:10.1371/journal.pone.0129841)
Supplement: S4 Table — (PDF) [file pone.0129841.s004.pdf]

DENSITY

MORPH	COL	DENSITY
BRANCHING	371	32
BRANCHING	372	33
BRANCHING	373	38
BRANCHING	374	36
BRANCHING	375	36
BRANCHING	376	30
BRANCHING	377	30
BRANCHING	378	34
BRANCHING	379	36
BRANCHING	380	38
MASSIVE	391	33
MASSIVE	392	37
MASSIVE	393	34
MASSIVE	394	34
MASSIVE	395	34
MASSIVE	396	35
MASSIVE	397	34
MASSIVE	398	36
MASSIVE	399	32
MASSIVE	400	30
**BR Mean**		**34.3**
**SD**		**2.983286778**
**SE**		**0.943398113**
**MA Mean**		**33.9**
**SD**		**1.969207398**
**SE**		**0.622718056**

DIAMETER

MORPH	COL	DIAM,cm
BRANCHING	371	0.113
BRANCHING	371	0.102
BRANCHING	371	0.123
BRANCHING	371	0.117
BRANCHING	371	0.127
BRANCHING	371	0.1
BRANCHING	371	0.095
BRANCHING	371	0.103
BRANCHING	371	0.128
BRANCHING	371	0.109
BRANCHING	371	0.127
BRANCHING	371	0.13
BRANCHING	371	0.127
BRANCHING	371	0.116
BRANCHING	371	0.12
BRANCHING	371	0.094
BRANCHING	371	0.1
BRANCHING	371	0.124
BRANCHING	371	0.126
BRANCHING	371	0.117
BRANCHING	372	0.115
BRANCHING	372	0.116
BRANCHING	372	0.115
BRANCHING	372	0.13
BRANCHING	372	0.124
BRANCHING	372	0.118
BRANCHING	372	0.118
BRANCHING	372	0.128
BRANCHING	372	0.116
BRANCHING	372	0.126
BRANCHING	372	0.126
BRANCHING	372	0.098
BRANCHING	372	0.127
BRANCHING	372	0.105
BRANCHING	372	0.113
BRANCHING	372	0.129
BRANCHING	372	0.131
BRANCHING	372	0.098
BRANCHING	372	0.101
BRANCHING	372	0.117
BRANCHING	373	0.117
BRANCHING	373	0.118
BRANCHING	373	0.123
BRANCHING	373	0.113
BRANCHING	373	0.13
BRANCHING	373	0.104
BRANCHING	373	0.096
BRANCHING	373	0.119
BRANCHING	373	0.133
BRANCHING	373	0.124
BRANCHING	373	0.106
BRANCHING	373	0.1
BRANCHING	373	0.095
BRANCHING	373	0.118
BRANCHING	373	0.114

MEAN DIAMETER

MORPH	COL	DIAM, cm
BRANCHING	371	0.1149
BRANCHING	372	0.11755
BRANCHING	373	0.11295
BRANCHING	374	0.11215
BRANCHING	375	0.1166
BRANCHING	376	0.11995
BRANCHING	377	0.11175
BRANCHING	378	0.11155
BRANCHING	379	0.11885
BRANCHING	380	0.11575
MASSIVE	391	0.11645
MASSIVE	392	0.11865
MASSIVE	393	0.1174
MASSIVE	394	0.1117
MASSIVE	395	0.1175
MASSIVE	396	0.1173
MASSIVE	397	0.114
MASSIVE	398	0.11125
MASSIVE	399	0.1147
MASSIVE	400	0.1136
**BRMean**		**0.1152**
**SD**		**0.010688368**
**SE**		**0.000755782**
**MA Mean**		**0.115255**
**SD**		**0.009519088**
**SE**		**0.000673101**

BRANCHING	373	0.09
BRANCHING	373	0.105
BRANCHING	373	0.123
BRANCHING	373	0.101
BRANCHING	373	0.13
BRANCHING	374	0.114
BRANCHING	374	0.093
BRANCHING	374	0.116
BRANCHING	374	0.092
BRANCHING	374	0.126
BRANCHING	374	0.126
BRANCHING	374	0.104
BRANCHING	374	0.111
BRANCHING	374	0.108
BRANCHING	374	0.107
BRANCHING	374	0.115
BRANCHING	374	0.115
BRANCHING	374	0.1
BRANCHING	374	0.112
BRANCHING	374	0.105
BRANCHING	374	0.13
BRANCHING	374	0.125
BRANCHING	374	0.113
BRANCHING	374	0.118
BRANCHING	374	0.113
BRANCHING	375	0.128
BRANCHING	375	0.107
BRANCHING	375	0.124
BRANCHING	375	0.131
BRANCHING	375	0.116
BRANCHING	375	0.118
BRANCHING	375	0.113
BRANCHING	375	0.091
BRANCHING	375	0.114
BRANCHING	375	0.119
BRANCHING	375	0.101
BRANCHING	375	0.108
BRANCHING	375	0.131
BRANCHING	375	0.129
BRANCHING	375	0.114
BRANCHING	375	0.119
BRANCHING	375	0.114
BRANCHING	375	0.129
BRANCHING	375	0.121
BRANCHING	375	0.105
BRANCHING	377	0.118
BRANCHING	377	0.119
BRANCHING	377	0.119
BRANCHING	377	0.122
BRANCHING	377	0.126
BRANCHING	377	0.126
BRANCHING	377	0.124
BRANCHING	377	0.129
BRANCHING	377	0.112
BRANCHING	377	0.122
BRANCHING	377	0.134
BRANCHING	377	0.114
BRANCHING	377	0.124

BRANCHING	377	0.115
BRANCHING	377	0.125
BRANCHING	377	0.105
BRANCHING	377	0.124
BRANCHING	377	0.114
BRANCHING	377	0.114
BRANCHING	377	0.113
BRANCHING	377	0.11
BRANCHING	377	0.102
BRANCHING	377	0.092
BRANCHING	377	0.119
BRANCHING	377	0.119
BRANCHING	377	0.101
BRANCHING	377	0.102
BRANCHING	377	0.123
BRANCHING	377	0.122
BRANCHING	377	0.125
BRANCHING	377	0.093
BRANCHING	377	0.13
BRANCHING	377	0.116
BRANCHING	377	0.102
BRANCHING	377	0.121
BRANCHING	377	0.096
BRANCHING	377	0.122
BRANCHING	377	0.104
BRANCHING	377	0.119
BRANCHING	377	0.117
BRANCHING	378	0.105
BRANCHING	378	0.13
BRANCHING	378	0.121
BRANCHING	378	0.116
BRANCHING	378	0.093
BRANCHING	378	0.109
BRANCHING	378	0.099
BRANCHING	378	0.107
BRANCHING	378	0.117
BRANCHING	378	0.118
BRANCHING	378	0.112
BRANCHING	378	0.103
BRANCHING	378	0.126
BRANCHING	378	0.102
BRANCHING	378	0.107
BRANCHING	378	0.121
BRANCHING	378	0.103
BRANCHING	378	0.116
BRANCHING	378	0.119
BRANCHING	378	0.107
BRANCHING	379	0.12
BRANCHING	379	0.114
BRANCHING	379	0.122
BRANCHING	379	0.118
BRANCHING	379	0.123
BRANCHING	379	0.126
BRANCHING	379	0.116
BRANCHING	379	0.129
BRANCHING	379	0.123
BRANCHING	379	0.101
BRANCHING	379	0.111

BRANCHING	379	0.11
BRANCHING	379	0.115
BRANCHING	379	0.127
BRANCHING	379	0.125
BRANCHING	379	0.126
BRANCHING	379	0.124
BRANCHING	379	0.118
BRANCHING	379	0.114
BRANCHING	379	0.115
BRANCHING	380	0.125
BRANCHING	380	0.107
BRANCHING	380	0.13
BRANCHING	380	0.132
BRANCHING	380	0.121
BRANCHING	380	0.126
BRANCHING	380	0.096
BRANCHING	380	0.131
BRANCHING	380	0.105
BRANCHING	380	0.093
BRANCHING	380	0.125
BRANCHING	380	0.114
BRANCHING	380	0.113
BRANCHING	380	0.126
BRANCHING	380	0.121
BRANCHING	380	0.113
BRANCHING	380	0.096
BRANCHING	380	0.105
BRANCHING	380	0.114
BRANCHING	380	0.122
MASSIVE	391	0.128
MASSIVE	391	0.119
MASSIVE	391	0.112
MASSIVE	391	0.117
MASSIVE	391	0.13
MASSIVE	391	0.118
MASSIVE	391	0.12
MASSIVE	391	0.117
MASSIVE	391	0.114
MASSIVE	391	0.122
MASSIVE	391	0.131
MASSIVE	391	0.102
MASSIVE	391	0.107
MASSIVE	391	0.105
MASSIVE	391	0.112
MASSIVE	391	0.112
MASSIVE	391	0.117
MASSIVE	391	0.119
MASSIVE	391	0.126
MASSIVE	391	0.101
MASSIVE	392	0.126
MASSIVE	392	0.113
MASSIVE	392	0.127
MASSIVE	392	0.112
MASSIVE	392	0.128
MASSIVE	392	0.122
MASSIVE	392	0.121
MASSIVE	392	0.128
MASSIVE	392	0.102

MASSIVE	392	0.11
MASSIVE	392	0.113
MASSIVE	392	0.111
MASSIVE	392	0.113
MASSIVE	392	0.106
MASSIVE	392	0.113
MASSIVE	392	0.131
MASSIVE	392	0.125
MASSIVE	392	0.123
MASSIVE	392	0.131
MASSIVE	392	0.118
MASSIVE	393	0.131
MASSIVE	393	0.118
MASSIVE	393	0.132
MASSIVE	393	0.11
MASSIVE	393	0.112
MASSIVE	393	0.127
MASSIVE	393	0.117
MASSIVE	393	0.118
MASSIVE	393	0.12
MASSIVE	393	0.113
MASSIVE	393	0.127
MASSIVE	393	0.127
MASSIVE	393	0.123
MASSIVE	393	0.122
MASSIVE	393	0.121
MASSIVE	393	0.128
MASSIVE	393	0.105
MASSIVE	393	0.098
MASSIVE	393	0.099
MASSIVE	393	0.1
MASSIVE	394	0.116
MASSIVE	394	0.105
MASSIVE	394	0.109
MASSIVE	394	0.112
MASSIVE	394	0.109
MASSIVE	394	0.125
MASSIVE	394	0.111
MASSIVE	394	0.113
MASSIVE	394	0.114
MASSIVE	394	0.125
MASSIVE	394	0.114
MASSIVE	394	0.119
MASSIVE	394	0.107
MASSIVE	394	0.097
MASSIVE	394	0.097
MASSIVE	394	0.107
MASSIVE	394	0.111
MASSIVE	394	0.096
MASSIVE	394	0.125
MASSIVE	394	0.122
MASSIVE	395	0.119
MASSIVE	395	0.115
MASSIVE	395	0.098
MASSIVE	395	0.127
MASSIVE	395	0.113
MASSIVE	395	0.123
MASSIVE	395	0.127

MASSIVE	395	0.123
MASSIVE	395	0.123
MASSIVE	395	0.125
MASSIVE	395	0.099
MASSIVE	395	0.117
MASSIVE	395	0.112
MASSIVE	395	0.108
MASSIVE	395	0.123
MASSIVE	395	0.125
MASSIVE	395	0.121
MASSIVE	395	0.112
MASSIVE	395	0.123
MASSIVE	395	0.117
MASSIVE	396	0.117
MASSIVE	396	0.111
MASSIVE	396	0.099
MASSIVE	396	0.122
MASSIVE	396	0.106
MASSIVE	396	0.131
MASSIVE	396	0.124
MASSIVE	396	0.124
MASSIVE	396	0.103
MASSIVE	396	0.124
MASSIVE	396	0.124
MASSIVE	396	0.117
MASSIVE	396	0.113
MASSIVE	396	0.128
MASSIVE	396	0.116
MASSIVE	396	0.113
MASSIVE	396	0.126
MASSIVE	396	0.102
MASSIVE	396	0.131
MASSIVE	396	0.115
MASSIVE	397	0.11
MASSIVE	397	0.095
MASSIVE	397	0.1
MASSIVE	397	0.116
MASSIVE	397	0.113
MASSIVE	397	0.105
MASSIVE	397	0.118
MASSIVE	397	0.128
MASSIVE	397	0.101
MASSIVE	397	0.118
MASSIVE	397	0.125
MASSIVE	397	0.119
MASSIVE	397	0.119
MASSIVE	397	0.115
MASSIVE	397	0.107
MASSIVE	397	0.112
MASSIVE	397	0.123
MASSIVE	397	0.12
MASSIVE	397	0.116
MASSIVE	397	0.12
MASSIVE	399	0.103
MASSIVE	399	0.099
MASSIVE	399	0.103
MASSIVE	399	0.115
MASSIVE	399	0.105

MASSIVE	399	0.104
MASSIVE	399	0.107
MASSIVE	399	0.123
MASSIVE	399	0.13
MASSIVE	399	0.131
MASSIVE	399	0.122
MASSIVE	399	0.11
MASSIVE	399	0.119
MASSIVE	399	0.099
MASSIVE	399	0.102
MASSIVE	399	0.115
MASSIVE	399	0.101
MASSIVE	399	0.103
MASSIVE	399	0.108
MASSIVE	399	0.126
MASSIVE	399	0.104
MASSIVE	399	0.107
MASSIVE	399	0.104
MASSIVE	399	0.111
MASSIVE	399	0.113
MASSIVE	399	0.121
MASSIVE	399	0.106
MASSIVE	399	0.102
MASSIVE	399	0.13
MASSIVE	399	0.125
MASSIVE	399	0.13
MASSIVE	399	0.125
MASSIVE	399	0.109
MASSIVE	399	0.11
MASSIVE	399	0.099
MASSIVE	399	0.123
MASSIVE	399	0.103
MASSIVE	399	0.121
MASSIVE	399	0.123
MASSIVE	399	0.128
MASSIVE	400	0.128
MASSIVE	400	0.112
MASSIVE	400	0.115
MASSIVE	400	0.117
MASSIVE	400	0.105
MASSIVE	400	0.119
MASSIVE	400	0.125
MASSIVE	400	0.107
MASSIVE	400	0.122
MASSIVE	400	0.128
MASSIVE	400	0.107
MASSIVE	400	0.122
MASSIVE	400	0.099
MASSIVE	400	0.106
MASSIVE	400	0.114
MASSIVE	400	0.124
MASSIVE	400	0.113
MASSIVE	400	0.102
MASSIVE	400	0.107
MASSIVE	400	0.1
**MEAN**		**0.115**
**Max**		**0.134**
**Min**		**0.090**

**SD**	**0.010**
**SE**	**0.001**
