# Supplementary material for: Contrasting Lesion Dynamics of White Syndrome among the scleractinian corals Porites spp
Source: PLoS One. 2015 Jun 29;10(6):e0129841. doi: 10.1371/journal.pone.0129841 (PMC4488276; doi:10.1371/journal.pone.0129841)
Supplement: S5 Table — (PDF) [file pone.0129841.s005.pdf]

**Multiple Sites WS prevalence; baseline surveys, 2006-2009**

| Site | Prev Pcyl | Prev P lob/lat | SE Pcyl | SE Plob/lat |
|---|---|---|---|---|
| **Achang** | | 1.95 | NA | 1.10 |
| **Anae** | 0.00 | 15.50 | 0.00 | 9.70 |
| **Cocos Lagoon** | 25.60 | 0.00 | 25.60 | 0.00 |
| **Fouha** | 0.00 | 0.00 | 0.00 | 0.00 |
| **Inner Double Reef** | | 1.85 | | 0.62 |
| **Outer Double Reef** | | 0.63 | | 0.63 |
| **Ipan** | | 22.10 | | 6.30 |
| **Luminao** | 9.24 | 17.78 | 3.93 | 9.69 |
| **Pago** | | 0.00 | | |
| **Pati Point** | | 0.00 | | |
| **Shark Pit** | | 6.70 | | |
| **Tumon Outrigger** | 9.00 | 5.10 | 2.40 | 5.10 |
| **Tumon outer reef** | | 2.80 | | 0.27 |
| **Tumon Ypao** | 0.00 | 0.00 | | |
| **Tupalao** | | 1.60 | | 1.60 |
| **Western Shoals** | 3.20 | 5.40 | 3.20 | 2.80 |
| **West Agana** | | 6.67 | | 6.67 |
| **Piti** | 38.49 | | 7.76 | |
| **Tanguisson** | 0.00 | 19.44 | 0.00 | 10.02 |
| **Adelup** | | 7.29 | | 1.62 |
| **Haputo** | 0.00 | 0.00 | 0.00 | 0.00 |
| **MEAN** | 8.55 | 5.74 | 4.19 | 1.61 |

*absence of value means species was not present at the site
